# Physicians’ Online Writing Language Style and Patient Satisfaction: The Mediator of Depth of Physician–Patient Interactions

**DOI:** 10.3390/healthcare11111569

**Published:** 2023-05-26

**Authors:** Jingfang Liu, Huihong Jiang, Shiqi Wang

**Affiliations:** School of Management, Shanghai University, Shanghai 201800, China; jingfangliu@shu.edu.cn (J.L.);

**Keywords:** depth of online interactions, patient satisfaction, online health counseling, online writing language style

## Abstract

Online health counseling (OHC) is increasingly important in modern healthcare. This development has attracted considerable attention from researchers. However, the reality of the lack of physician–patient communication and dissatisfaction with online health services remains prevalent, and more research is needed to raise awareness about important issues related to OHC services, especially in terms of patient satisfaction and depth of interaction (i.e., the product of the number of interactions and the relevance of the content). This study constructs an empirical model to explore the relationship between physicians’ online writing language style (inclusive language and emojis), depth of physician–patient interactions, and patient satisfaction. The study obtained 5064 online health counseling records from 337 pediatricians and analyzed them using text mining and empirical methods. The results showed that physicians’ inclusive language (β = 0.3198, *p* < 0.05) and emojis (β = 0.6059, *p* < 0.01) had a positive impact on patient satisfaction. In addition, the depth of the physician–patient interaction partially mediated this effect. This study promotes a better understanding of the mechanisms of physician–patient interactions in online settings and has important implications for how online physicians and platforms can better provide online healthcare services.

## 1. Introduction

### 1.1. Background

Internet technologies are changing the way how people provide, access, and disseminate health management knowledge with many conveniences [1,2]. Online health counseling, one of the important ways for people to access health information in this context, refers to the transfer of traditional offline, face-to-face interaction to instant online interaction. It aims to link the potential “supply” (physicians) and “demand” (patients) markets through virtual networks and to realize value by reducing the cost and improving the accessibility of healthcare services [3]. Unlike previous offline consultations, this new counseling model not only provides patients with more choices but also allows them to leave feedback on the platform about their “satisfaction” or “dissatisfaction” with the physician’s services after the counseling is completed [4]. Previous studies have shown that these feedbacks directly reflect the quality of the physician’s online services and are also related to better health outcomes, rapport, and trust [5,6]. Therefore, in order to realize the value and rewards of online healthcare, it is a very important question how online physicians can provide their patients with satisfactory services based on this novel approach.

The term patient satisfaction refers to the level of patient satisfaction with the services provided by the physician. Previous studies have examined the influencing factors related to patient satisfaction, mainly from interactions and the verbal content of the physician. For example, Chen et al. extracted informational support and emotional support from online physician–patient interaction texts and confirmed their positive impact on patient satisfaction [4], while Yang et al. found that frequency of interaction and responsiveness in physician–patient interactions were equally important aspects of patient satisfaction through an exploration of online physician service delivery processes [5]. However, although the literature on online physician discourse and interaction has contributed to our understanding of physician–patient interactions, the current study is limited in at least two ways:

First, the current study explored limited aspects of physicians’ linguistic features. In the context of online health counseling, the services provided by physicians are mainly represented through texts [7]. Among the essential elements of these texts, language shapes not only the content of the message (i.e., what is said?) but also the style (i.e., how is it said?). However, most prior research has focused on the informational content of physicians’ linguistic features [8,9], and there are rich linguistic features (e.g., psychologically relevant aspects of language style and the use of paralanguage) that have not yet received attention. From the perspective of linguistic expectancy theory, to increase patient satisfaction, physicians’ linguistic features must match patients’ expectations [10,11]. However, due to the heterogeneous nature of online counseling services, patients’ expectations are often vague and difficult to grasp [5]. Therefore, online physicians need extra efforts to compensate for the ambiguity of these expectations. We suggest that online physicians do this through the tool of linguistic style, which is more closely connected to a person’s psychological world than to linguistic content [12]. Although the linguistic style is not usually consciously manipulated by people, it is an important aspect of social influence that may become part of a physician’s efforts [13]. There is reason to believe that well-structured textual content can create an atmosphere that brings physicians closer to their patients and makes them more understandable, thus increasing patient satisfaction.

Second, multiple independent studies in multiple fields have focused on the direct effects of language style [11,14,15,16] and interaction [5,8,17] on communication outcomes, yet few studies have addressed the mechanisms of influence between them. This work explored the effect of physician writing language style on patient satisfaction and the mediating role of the depth of physician–patient interaction. The depth of physician–patient interaction is defined as interactions closely related to the topic, as interactions with clear goals are more likely to stimulate in-depth discussions [18]. Although previous research has identified the frequency of physician–patient interactions as an important aspect of patient satisfaction [5,8], not all interactions are relevant in practice. Therefore, patients are unlikely to focus only on the frequency of interactions [9]: it is necessary to consider the depth of physician–patient online interactions. In addition, from the perspective of information dissemination, the linguistic style of online writing gives more substance to interpreting information as an important complement to the absence of cues in the online interaction environment. Although these linguistic features are often unconscious behaviors, they reveal more important aspects of interpersonal interactions, such as deception and trustworthiness [19,20]. It is well documented that people judge others by their linguistic style, such as friendliness and ease of getting along [21,22]. Therefore, the language style of physicians’ online writing inevitably affects the depth of interaction and patient satisfaction.

### 1.2. Research Questions

To address this research gap. This study focuses on two basic language styles in physicians’ online writing: (1) inclusive language. This refers to using words related to the group (e.g., we) to reflect concern for others; (2) Emojis. This is a textual paralanguage used to convey emotion and encouragement.

We chose these two language styles for several reasons: first, focusing on the distance between the physician and the patient is essential [23]. In previous studies, inclusive language and emoticons are two language styles associated with psychological distance [11,24]. Through these two languages, patients can capture how the physician treats them (to determine whether they are on equal footing with the physician in the communication) and the physician’s emotional state (to assess the physician’s mood when providing services) [25,26]. Therefore, when it comes to these two aspects, physicians must be aware of their linguistic expressions. Second, inclusive language represents the linguistic elements of physicians’ discourse, and emoticons represent the paralinguistic elements. They capture two of the most critical components of the online doctor-patient interaction environment [9,27]. Finally, although other writing language styles, such as lexical richness and text length, can be chosen, they are unrelated to psychological distance. In summary, we planned to answer the following research questions:

RQ1: Does a physician’s online writing language style (inclusive language and emojis) affect patient satisfaction? If so, how does this occur?

RQ2: How does the depth of physician–patient interaction play in the relationship between physician online writing language style and patient satisfaction?

To answer the above research questions, we critically reviewed the existing literature on physician–patient online interactions to identify research gaps. Immediately afterward, we proposed a research model for this paper that demonstrates an operational mechanism for the effect of physicians’ online writing language style on ultimate patient satisfaction. We then test our model and theory by collecting objective online counseling records from a mobile health platform. Finally, we discuss the implications and limitations of this study.

In summary, this study will demonstrate the impact mechanism of online writing language style on patient satisfaction, providing a new perspective for a deeper understanding of online physician–patient interaction. The results of this study will help to reveal how to improve doctors’ service delivery behavior and platform management to enhance the effectiveness of online physician–patient interaction.

## 2. Theoretical Background and Literature Review

This study conducted a literature search using the “Web of Science,” focusing mainly on literature from the past decade. To ensure the quality of the searched literature, we also set the search to obtain only SCI and SSCI-related literature from the “Web of Science Core Collection”.

### 2.1. Online Pediatric Health Counseling

Online health counseling is one of the most important parts of online healthcare and is the principal place where online physicians and patients get to know each other online. Online physicians diagnose primarily through virtual information such as text and patient pictures. The provision of healthcare services through the Internet has proven to be uniquely advantageous. It can significantly reduce the cost of medical services, alleviate the imbalance in the distribution of medical resources due to geographical factors, and effectively improve the efficiency of disease interventions [3]. Therefore, this Internet-based platform for healthcare services represents a more convenient resource.

Pediatrics is unique and representative of many departments with online health counseling. The number of visits to online pediatrics is enormous [9]. Unlike other departments, the users consulting in pediatrics are often not the patients themselves but their guardians. Users are more likely to express dissatisfaction and create interpersonal conflicts with pediatricians out of concern and strong emotions for their loved ones [28]. A series of studies have shown that pediatricians need to pay special attention to communication with their patients, for example, by paying attention to the expression of their language [29] and the expectations of the patient [30,31]. Therefore, it is essential to understand the factors influencing online pediatric patient satisfaction based on the perspective of the doctor–patient interaction.

### 2.2. Physician Language Characteristics and Patient Satisfaction

In the health communication literature, an association between physicians’ linguistic characteristics and patient satisfaction has been established. We have reviewed relevant research in the past decade and summarized representative literature in Table 1. Overall, a series of studies suggest that physicians’ verbal content is a key element influencing patient satisfaction [8,9]. Moreover, verbal expressions have received much attention recently. In a limited number of studies, researchers have noted the potential value of physicians’ online verbal expressions [7,27,32], but few studies have considered both verbal and paralinguistic aspects. However, linguistics and paralinguistics are important aspects of online writing language style [33]. From the perspective of information dissemination, paralanguage serves as an important complement to compensate for the absence of non-linguistic cues in online interactive environments, giving more connotations to information interpretation. Based on the above analysis, we analyzed the linguistic and paralinguistic aspects of physicians’ online writing language styles regarding their impact on patient satisfaction.

### 2.3. Linguistic Expectancy Theory and Psychological Distance

One potential theoretical explanation for why physicians’ online writing language style affects patient satisfaction is the Language Expectancy Theory (LET), a linguistic theory focusing on informational features that explains how linguistic features (e.g., word choice, language style, etc.) act on the outcome of communication [10,11]. The theory argues that based on certain social and cultural norms, people develop expectations about the appropriate communication style chosen by their communication partners. Linguistic features during interactions can positively or negatively violate linguistic expectations. If the speaker’s linguistic features exceed expectations, a positive violation occurs, facilitating communication. Conversely, if the speaker negatively violates linguistic expectations, negative violations can occur, which can negatively impact communication outcomes.

Previous literature has identified language styles associated with expectations, often related to psychological distance [11,14,15]. Psychological distance refers to how far people perceive things from them temporally, spatially, socially, and hypothetically [35]. Psychology-related literature suggests that people’s processing of information (i.e., the way they encode or decode information) changes systematically with their perception of the psychological distance of a message, thus affecting the assessment of its authenticity [36,37]. When the mental distance is distant, people usually abstractly understand messages, presenting greater uncertainty. In contrast, people tend to think more concretely when mental distance is close, leading to more engagement.

In online health consultations, physicians and patients tend to be psychologically distant due to power relations. Although some physicians and patients establish contact early on or offline, most online physicians and patients do not know each other [8]. The long distance creates more uncertainty for online consultations. Maristella, in a study examining disease treatment adherence, called for physicians to focus on reducing the psychological distance between them and their patients to promote communication and trust [23]. Thus, according to LET, we can examine how physicians’ online writing language style affects patient satisfaction, which may have a practical impact on enhancing physician–patient communication, improving the physician–patient relationship, promoting medical practice, and reducing uncertainty [38,39,40].

### 2.4. Depth of Online Interactions and Physician-Patient Relationship

As a result of the level of interaction, this study defines the depth of online interaction as the product of the number of interactions and the correlation between the content of the interaction. However, this definition is different from previous literature research in healthcare [8]. We summarize representative studies related to the depth of interaction over the past decade in Table 2. Overall, the depth of interaction was initially understood as the number of interactions, as more interaction often means more participation [8,41]. However, researchers are gradually realizing that content is also an aspect that cannot be ignored [9]. Yoon et al. recently developed a framework for interactivity analysis, which considers targeted and content-related interactions as critical avenues for in-depth interaction [18]. Due to the unique nature of online interaction scenarios, although the number of interactions is essential for patients [5,8], multiple interactions do not necessarily represent in-depth communication between the two parties. Multiple interactions may occur if patients and physicians cannot interact clearly or understand each other. Therefore, it is necessary to consider the penalty term for the number of interactions from the content perspective to extract the content-related parts of multiple interactions between physicians and patients.

We also tend to understand the depth of interaction from the perspective of the doctor–patient relationship. Researchers argue that people build social bonds through a series of interactions. The establishment of this social connection has a certain degree. Therefore, interpersonal relationships are assessed as “weak relationships” and “strong relationships” [42]. A “weak relationship” is usually a relationship that forms quickly and does not require much commitment. In contrast, a strong relationship tends to imply more emotions and resources and is considered to be more accessible to knowledge [43]. Moreover, the dynamic nature of social bonds cannot be ignored, as “weak relationships” can develop into “strong relationships” through communication [44,45]. In this context, the depth of interaction and establishing strong relationships are very relevant. In a typical online health consultation scenario, the doctor assesses the patient’s health status by asking questions and obtaining information from the patient’s queries. When a patient consults a physician through an OHC platform, the beginning of a new relationship shows high uncertainty. As the physician and patient discuss their concerns, the relationship evolves from surface-level communication to deeper communication. In the “strong relationship” scale developed by Guo et al. between physicians and patients, frequency of interaction and topic content are key reference dimensions [44].

Therefore, based on the discussion of the relevant literature, the depth of interaction defined in this study considers the number of interactions and the content.

## 3. Research Model and Hypotheses

### 3.1. The Effect of Physician Writing Language Style on Patient Satisfaction

Inclusive language is an other-oriented language style [11]. In a psychosocial health study, Mascaro et al. found that inclusive language helped convey empathy and support and reduced patient depression [46]. Furthermore, in clinical research, Caitlyn et al. also found that successful clinicians demonstrated humble behaviors by using more inclusive pronouns (e.g., we) rather than exclusive words (e.g., I) and empathy-rich expressions that facilitated patient trust and health management [47]. Despite the importance of disease-related information in online health consultations, many recent studies on patient–physician online interactions point out that attention to patient emotions is equally important and should be given a higher priority [4]. Therefore, according to language expectancy theory, we consider inclusive language as a positive violation. This is because inclusive language conveys additional empathy and support. Therefore, we hypothesize that:

**H1:**  *The effect of physicians’ inclusive language on patient satisfaction is positive.*

Another physician’s writing language style in this study was emoji. Emoticons are a textual paralanguage often considered a vehicle for emotions. In a study on the usefulness of consumer comments, Wu et al. found that comments that included emojis elicited more positive emotions and received more likes and retweets than comments that did not include emojis [19]. Furthermore, given the growing importance of emojis in online interactions, Ge et al. propose emojis as an emerging rhetorical device to adapt to changing communication norms [48]. This is a significant change for scholars in the online health field, and using emojis may be an excellent opportunity to take physician–patient communication to another level. For example, scholars such as Skiba focused on the unique meaning of emojis in healthcare and attempted to create a unique set of emojis to integrate into everyday medical practice [49]. Compared to the role of emoticons, emojis not only capture readers’ attention, improve their understanding of text content, and convey emotion but are richer in presentation and content [50]. In time-critical online healthcare environments, emoticons can even facilitate important clinical decisions. Therefore, based on linguistic expectancy theory, we consider emojis as a positive violation in physician-patient interaction scenarios, and we hypothesize that:

**H2:**  *The effect of physicians’ use of emojis on patient satisfaction is positive.*

### 3.2. The Mediating Effect of Depth of Interaction

The depth of interaction expresses a deep connection. The deeper the connection between them when there are more content-related interactions. However, topic drifts in online health communities are a common phenomenon. Park et al. studied the negative effects of topic drift in online health community discussions on communication experiences and relationship building. They found that discussion members became frustrated [51]. As a result, patients are more likely to benefit from semantically relevant discussions. When the depth of interaction reaches a deeper level, this physician–patient bond is stronger. Patients trust the physician more and are willing to give positive feedback about the relationship, such as likes, positive comments, and gifts. Therefore, we hypothesize that:

**H3:**  *The effect of the depth of online interaction on patient satisfaction is positive.*

To avoid topic drift, previous literature has focused on the role of psychological distance in how the interacting parties encode information [36,37]. This view suggests that when people interact at a psychological distance, they tend to understand the information abstractly and discuss topics that tend to be more macro and casual, and conversely, when psychological distance is close, people understand the information more concretely.

Prior evidence suggests that shaping the psychological distance between interacting parties can be operationalized through the strategic use of language [52,53]. For example, Nishimura et al. examined the psychological distance function of honorific language and concluded that this linguistic phenomenon could have an impact on interpersonal relationships and interactions [54]. A language with a low psychological distance is more conducive to facilitating communication than a language with a high psychological distance [55]. In online health counseling scenarios, inclusive language (e.g., we) facilitates communication primarily by promoting cohesion and increasing the sense of interdependence among interacting parties. On a practical level, perception clearly differs between the physician’s expression “we can take medication” and “you can take medication”. This is because replacing “we” with “you” brings about a lack of emotional identity and a sense of distance [56].

In addition, emojis may play a more critical role in developing the relationship. As Ji et al., in a review, examined new applications of emojis in physician–patient interaction scenarios and proposed emojis as an effective tool to improve physician–patient communication [26]. A recent study of an online survey found that emojis can elicit positive emotions and enhance a brand’s social presence, thus bringing consumers closer to the brand [57]. In examining online information sharing, Jeon et al. suggested that when there is a greater sense of authenticity between the two parties of online interaction, they are more likely to interact deeply [24]. Thus, physicians’ linguistic strategies that reduce psychological distance help to understand the content of the message and thus facilitate physician–patient interaction. Therefore, we hypothesized that:

**H4:**  *The effect of physicians’ use of inclusive language on the depth of online interactions is positive.*

**H5:**  *The effect of physicians’ use of emojis on the depth of online interactions is positive.*

### 3.3. Research Model

**Figure 1:**
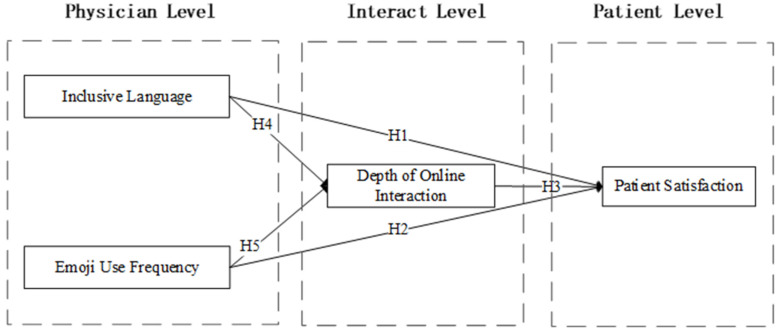
Research Model.

Finally, we constructed this multilevel research model (shown in Figure 1). In this study, physician-level inclusive language and emojis were identified as linguistic styles important for the depth of physician–patient online interaction at the interaction level and patient satisfaction at the patient level. In addition, we investigated the mediating role of the depth of physician–patient interaction on the linguistic style of physician online writing and patient satisfaction.

## 4. Methods

### 4.1. Data Collection and Preprocessing

**Figure 2:**
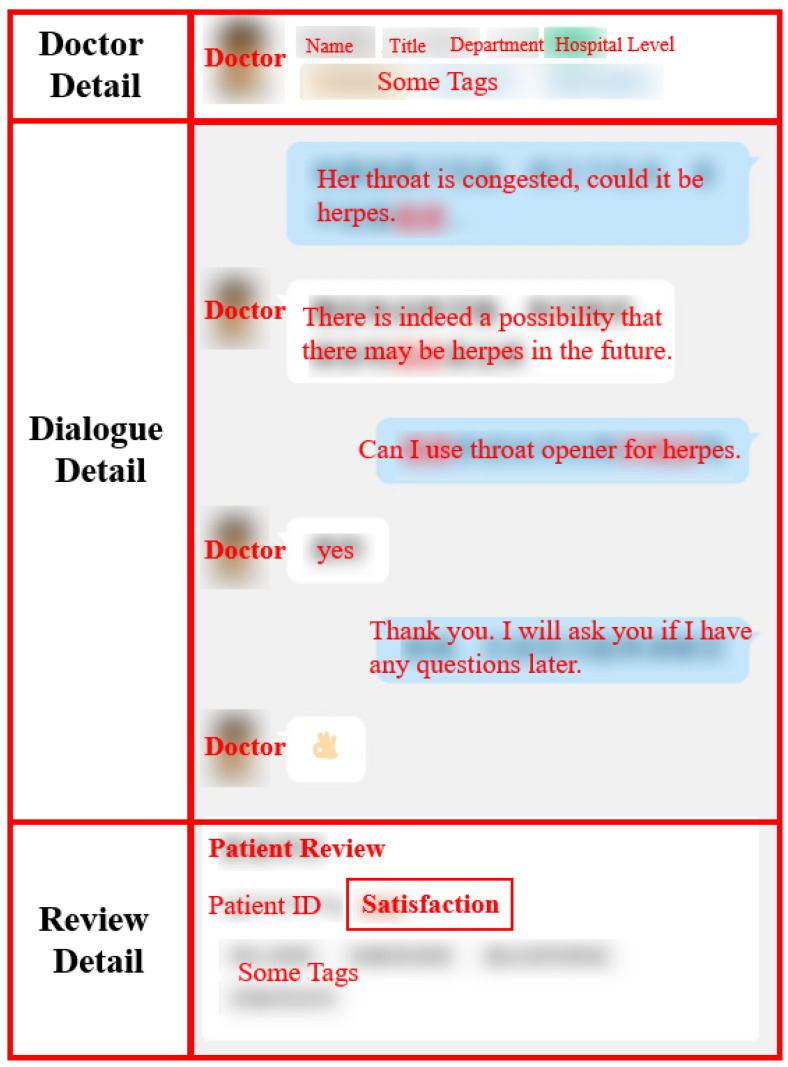
Research Data.

The Chunyu Physician app was selected as the data source for this study. The platform was established in July 2011 and is one of China’s largest mobile health communities. By 2020, Chunyu Physician had attracted 140 million users and over 630,000 public hospital practitioners and served over 400 million patients. As shown in Figure 2, We collected 360 days of online pediatric consultation records since 14 February 2022.

We selected the study dataset for this paper based on two criteria. (1) We obtained data from pediatrics only. In addition to the relevance of pediatrics to our research question and the abundance of data, another reason was that we wanted to construct a semantic space of online physician–patient interactions to quantify the relevance of the content of physician–patient interactions, and choosing specific departments helped to avoid additional noise. (2) We avoided invalid data from entering the dataset. We excluded query records containing no more than one round of interaction (i.e., only one question and one answer) to ensure the integrity of the interaction. In addition, we cleaned invalid text data (e.g., links and autoresponder text) because they do not provide valid information and can interfere with the results.

**Figure 3:**
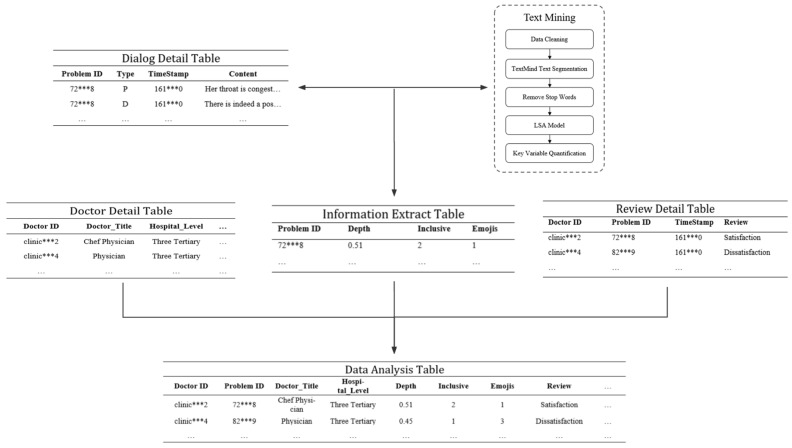
Data Pre-processing.

We analyzed and processed the dataset of this study under the guidance of the framework shown in Figure 3. For all processed data, we summarize and analyze it at the physician–patient interaction level. The final data analysis table included 5064 data records from 337 doctors.

### 4.2. Variable Design and Measurement

#### 4.2.1. Dependent Variable

The dependent variable in this paper is patient satisfaction. After counseling, patients can rate the physician’s service and leave feedback on the platform. If patients are satisfied with the physician’s online service, they mark the counseling as “Satisfactory”. We quantify patient satisfaction through rating labels, coding satisfaction as 1 and others as 0.

#### 4.2.2. Independent Variables

Inclusive language (Inclusive) and frequency of emoji usage (Emojis) are the independent variables in this paper. We extracted these linguistic features with the help of the TextMind tool [58]. Inclusive language is quantified as the sum of first-person plural pronouns (e.g., we) [11,59]. First-person plural pronouns bring the parties closer by expressing concern and emotional identification with others. It implies that the speaker and the receiver of the message are a community of interest and share a common goal. The frequency of emoji use is the sum of the number of emoji used by the physician in the interaction. We constructed an emojis lexicon to identify emojis in sentences.

#### 4.2.3. Mediating Variable

The mediating variable in this paper is the depth of the online interaction between the physician and the patient. Online counseling is usually composed of multiple physician–patient interactions, and although Yang et al. [8] used the number of interactions as an agent for interaction depth, as [9] showed, this measure is not sufficiently valid because not all interactions are content-related. Therefore, this study uses the content relevance of the physician’s text and the patient’s text to calculate the penalty term. The depth of the physician-patient online interaction is treated as the product of the content relevance (SemR) and the number of interactions (*n*):

(1) $$D\left(j\right)=n\times SemR,$$

where SemR is a probability value between 0 and 1, if the physician and patient interacts online 10 times, but their content relevance is only 0.8, then the depth of the interaction is 8. We used a technique called latent semantic analysis (LSA) to calculate SemR [60]. As a supplement, we describe the measurement and data processing in detail in Appendix A.

#### 4.2.4. Control Variables

We added a set of control variables to avoid the endogeneity problem of the model. First, the quality of the physician’s service is an important aspect of the service content, and we controlled for indicators regarding the quality of the physician’s online service; they include the physician’s response speed (Rst) [5,9]; the physician’s recommendation index (GoodR), and the physician’s online counseling volume (Total). In addition, for physician gender (D_Gender), we coded male physicians as 1 and female physicians as 0. We also controlled for month–time fixed effects (Month) and individual physician fixed effects (Physician), considering individual differences in physician services and seasonal effects of diseases. Second, at the patient level, we included patient question complexity (QLen) in our study model [61], using the total number of words asked by the patient during the counseling, considering that the characteristics of the disease itself may influence the complexity of the question. Then, based on previous studies [9,32], we also included patient initiative (P_Active) and patient politeness (P_Civility). Finally, the counseling price (price) may influence user interaction, so we included it in our model [9]. The definitions and metrics associated with the variables are shown in Table 3.

### 4.3. Descriptive Statistics and Correlation Analysis

To further understand the data in the model, descriptive statistical analysis was performed on the study variables. The data are noteworthy for the highly skewed distributions of online consultations with physicians (maximum 100708), counseling price (maximum 437), and complexity of patient questions (maximum 2147), so the variables were logarithmically processed. The descriptive statistics of the processed data are shown in Table 4.

Considering that severe multicollinearity problems in the study model can lead to large fluctuations in the estimated values of the regression coefficients and invalidate the significance test, we conducted correlation analysis on the study variables (Appendix D). The absolute values of the correlation coefficients between the independent variables are less than 0.5, which indicates that there is no multicollinearity problem in the model.

## 5. Results

### 5.1. Hypothesis Test

Since the mediating and dependent variables of the research model in this paper are continuous and dichotomous, we use logistic regression and the coefficient product method for hypothesis testing. Logistic regression can provide low variance coefficient estimates for categorical variables, and the coefficient product method can test the mediating effect when the dependent variable is a rank variable.

We constructed several models to test the hypotheses in this paper’s study (In Appendix B, we detailed the formula calculations of these models). Table 5 shows the OLS regression models of physician online writing language style on the depth of physician–patient online interaction. We constructed a baseline model and gradually added independent variables. In the first step, only the control variables were placed into Model 1; in the second step, the explanatory variable inclusive language was introduced to construct Model 2 based on Model 1; and in the third step, the explanatory variable emoji was added to Model 2. Model 3 is the complete model with all independent variables. The results of Model 2 and Model 3 show that the frequency of physicians’ emoji use (β = 0.3064, *p* < 0.01) and inclusive language (β = 1.3604, *p* < 0.01) have a significant positive effect on the depth of online interactions, supporting our research hypotheses H4 and H5.

Table 6 shows two logistic regression analysis models to test the effect of physician online writing language style and depth of interaction on patient satisfaction, respectively. In Model 1, the results show that the frequency of physicians’ use of emojis (β = 0.6059, *p* < 0.01) and inclusive language (β = 0.3198, *p* < 0.05) was positively associated with patient satisfaction. This suggests that physicians’ writing language style significantly increased patient satisfaction, supporting the hypotheses of H1 and H2. In Model 2, the results showed that depth of interaction positively and significantly affected patient satisfaction (β = 0.0575, *p* < 0.01), supporting our research hypothesis H3.

Further, we used the R package Remediation and tested for mediating effects according to the product distribution method, and calculated to obtain inclusive language → depth of interaction → patient satisfaction path with 95% confidence intervals of [0.038, 0.120]; and the frequency of emoji use → depth of interaction → patient satisfaction path with 95% confidence intervals of [0.008, 0.028]. These two confidence intervals did not include 0. Therefore, the mediating effect of interaction depth on language style and patient satisfaction was significant, further supporting our research hypothesis above. The results of the hypothesis testing are summarized in Table 7.

### 5.2. Robustness Checks

To further confirm the reliability and generalizability of the findings, we conducted robustness tests using two methods. First, the model may have missed unobserved patient characteristics that act on both physician writing style and interaction depth, leading to endogeneity issues. To alleviate this concern, this study used the average writing language style of physicians corresponding to all patient interactions in the month before the patient interaction as an instrumental variable (Inclusive_y, Emojis_y) because writing language style is typically an unconscious act [13] and is unlikely to change over a short period. In addition, this variable is unlikely to correlate with current patient characteristics, as patients are unlikely to perceive physician language style in previous patient interactions. The results are shown in Table 8, where the Cragg-Donald Wald F statistic values in the first segment of the regression results for both models (1) and (3) were greater than 16.38, and the hypothesis of a weak instrumental variable was rejected [62]. Meanwhile, the results in Table 8 are consistent with the above results, indicating that our study model is robust.

Second, this study ranked the samples according to the time of occurrence of the physician–patient interaction and regressed them after removing the top 5% and bottom 5% of the samples. The results are shown in Appendix C, and they are generally consistent with the above results. The robustness of the model was further demonstrated, suggesting that our findings are reliable.

### 5.3. Additional Analysis

In the above discussion, we found that a higher frequency of physician emoji use was associated with a higher depth of interaction and patient satisfaction. A potential problem, however, is that online health counseling is a highly professional-focused scenario, and excessive use of emojis by physicians is likely to reduce patients’ perceptions of their professionalism [63]. Therefore, we further investigated the non-monotonic effect of the impact of emoji production by adding a quadratic form of emoji usage frequency to the model.

**Figure 4:**
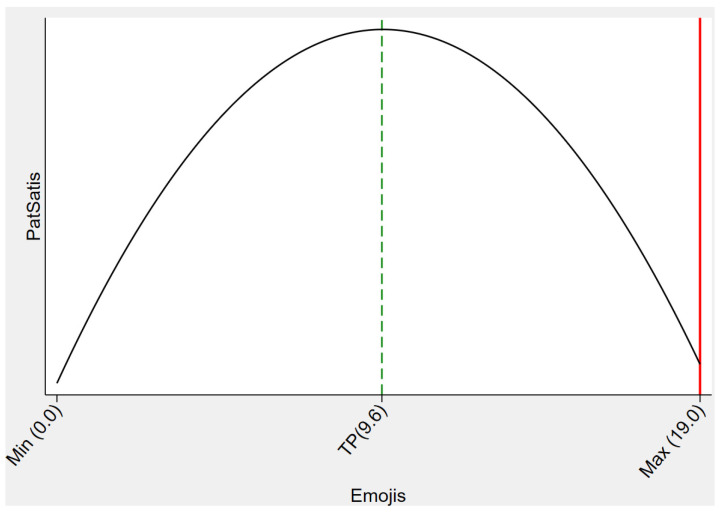
Inverted U-shaped relationship between emoji use and patient satisfaction.

The results in Table 9 indicate that the effect of physician emoji usage frequency on patient satisfaction is not a simple linear relationship but shows an inverted U-shaped curve (as shown in Figure 4). Further, we performed a test of the U-shaped curve according to [64], and the results pointed to a significant inverted U-shaped relationship with an extreme value point of 9.6 at the 5% level (confidence interval [7.087458, 16.534115], *p* = 0.012). That is, when physicians use emoticons more frequently than 9.6, it will decrease patient satisfaction.

## 6. Discussion

In summary, this study investigated the effect of physicians’ online writing language style on patient satisfaction based on linguistic expectancy theory and that this effect can be mediated through interaction depth. This topic is rarely addressed in the existing literature, and our findings reveal deeper reasons for the role of physicians’ language. Specifically, we hypothesized that the more inclusive language and emoticons physicians use in OHC, the higher the depth of physician–patient online interactions and patient satisfaction. We also hypothesized that the depth of physician–patient interaction mediates this relationship.

### 6.1. Key Findings

Based on our research questions and the rich data available on the platform, our analysis reveals several important findings.

First, we found that physicians’ online writing language styles (inclusive language and emoticons) are an important component of online health consultations, and they positively and significantly impact patient satisfaction. This suggests that, in addition to processing information content, attention to physicians’ online language style is a very important aspect of improving patient satisfaction. In addition, physicians’ use of emoticons had a greater impact on patient satisfaction and showed a non-linear effect, with an inverted U-shaped relationship between them. This suggests that, although the use of emoticons may trigger positive emotions, it may not be a good idea for physicians to use them extensively in interactions.

Second, Interaction depth has a significant mediating effect between physicians’ online writing language style and patient satisfaction. Our analysis shows that interaction depth matters to patients. Patients will be more likely to interact deeply with their physicians if they use more inclusive language and emoticons. This is because, in contrast to offline scenarios, consultations are mostly conducted for minor illnesses in online health consultations [65], where patients may have more psychological demands. Therefore, in addition to guiding the diagnosis of the condition and medication, physicians must reduce the psychological distance to calm patients and help them understand medical information. Language strategies that reduce psychological distance will help to enhance the focus of the interaction and facilitate communication between the two parties, which will lead to more effective health management knowledge and, thus, more patient satisfaction.

### 6.2. Theoretical Contributions

As the study to discuss the relationship between linguistic style, depth of physician–patient interaction, and patient satisfaction based on linguistic expectancy theory, our study provides theoretical contributions in several ways.

First, this work extends the application of theory and enriches the online health-related literature by applying language expectancy theory to online health consultations. Based on the perspective of language expectancy theory, we identify inclusive language and emojis in physicians’ online writing language styles in as-expected communication norms and test their effects on patient satisfaction. Unlike previous studies that have focused on content, this work focuses on writing language style. We also focus on both the linguistic and paralinguistic aspects of writing language style. This application will help provide new perspectives and thus new insights for improving patient satisfaction.

Second, our study identified a reliable new way physician writing language style affects patient satisfaction. Therefore, this study contributes to the pragmatics literature by further extending the theoretical model of linguistic expectations. Unlike previous studies that have mostly focused on the direct effects of linguistic style and communication outcomes [3], the present study discusses the mediating role of interaction depth. Based on a language expectancy theory perspective, our results confirm that physicians’ online writing language style significantly influences the depth of the physician–patient interaction and, consequently, patient satisfaction. This finding helps to expand the theory by adding new mediating paths to the linguistic expectancy theory model.

Third, this study contributes to the literature on physician–patient interactions in online health by discussing the antecedents of deep online physician–patient interactions. This is rarely involved in previous studies. Truly effective online interactions require not only a connection between online physicians and patients but also deep interactions to promote mutual understanding. In contrast to Yang et al.’s study [8], we considered the content of physician–patient interactions. Our analysis indicates that physicians’ online writing language style plays an important role in online patient–physician interactions, and when physicians’ language and wording bring both parties closer psychologically, physicians and patients show a higher depth of interaction and contribute to patient satisfaction. This exploration provides new empirical support for promoting effective online physician–patient interactions.

### 6.3. Practical Implications

This study also offers practical implications for physician practices and platform management.

First, physicians participating in online healthcare platforms can use our findings to improve the depth of online interactions with patients and patient satisfaction. According to our findings, physicians’ online writing language style (inclusive language and emoticons) positively improved the depth of physician–patient interactions. Psychological distance from the patient is a concern in online physician–patient interactions. If there is a high psychological distance, the patient’s needs may not be accurately identified. In that case, there is a high risk of misunderstanding, which negatively affects the online counseling’s effectiveness. Therefore, physicians should use low psychological distance language to help reassure patients and help them understand and receive medical information. Finally, our findings highlight the importance of physicians’ use of emoticons during patient–physician interactions, which helps patients understand and receive health information. A good physician should, first and foremost, be a good listener. Emojis can help alleviate communication barriers due to language and cultural differences, help physicians project an amiable image, and close the distance between them and their patients. This mechanism can be widely applied to the field of physician–patient online interaction.

Second, our findings provide insights into the IT design of OHC platforms. In this study, physicians’ online writing language style plays an important role in online physician–patient interactions. Our findings indicate that physicians and patients exhibit a higher depth of interaction and patient satisfaction. Physician–patient interactions are more effective when the physician’s language and wording meet expected communication norms. Therefore, the OHC platform can improve the normality of linguistic expressions by automatically detecting wording norms and linguistic cues in physicians’ online texts. Different language models can be trained for physicians, thus providing conversational cues for online physicians to edit text content and maximize the credibility of discourse. Moreover, in practice, we have noticed that physicians’ use of emojis helps them to interact deeply with patients. However, there are not many types of emojis that can be used in medical scenarios, as advocated by Skiba and other scholars [49]; the OHC platform can work on developing a set of emojis for medical scenarios to enrich the semantic representation of emojis in medicine, create a good interactive environment, and improve the depth of physician–patient interaction.

### 6.4. Limitations and Future Research

This study has several limitations and points to several directions for future research based on these limitations. First, this paper is based on a mobile counseling scenario in which most illnesses are minor. However, the severity of the illnesses may affect the depth of online interactions. Back in 1996, Ford et al. analyzed the content of 117 outpatient records and found that consultations for life-threatening diseases, such as cancer, were clinician-driven, leaving little room for patient discussion [66]. Has this improved in today’s mobile health applications? What is the depth of patient–physician interaction online? Therefore, further studies could collect data on patient–physician interactions in intensive care units by enriching our findings. Second, due to the privacy protection of the platform, some important patient characteristics (e.g., patient gender and educational background) were not available to us, and they may affect the depth of online interactions and patient satisfaction. Therefore, future studies could further extend our work if patient characteristics exist. Finally, online health consultations in pediatric departments are more often parental consultations, so future studies could replicate our study in the context of in-person patient consultations and help us further consolidate our findings.

## 7. Conclusions

The linguistic style of physicians’ writing is often overlooked in online health counseling scenarios. This study provides a new perspective based on linguistic expectancy theory, a research model that focuses on three levels of physician–patient interaction, that is, physician, interaction, and patient. We investigated how the linguistic and paralinguistic aspects of physicians’ online writing language styles (i.e., inclusive language and emoticons) affect patient satisfaction from the perspective of online writing language styles and explored new pathways for mediating interaction depth. The results suggest that online physicians’ online writing language style is key to improving patient satisfaction and facilitating deeper online communication between physicians and patients. We further analyzed the non-monotonic effects of physician emoji use. This work expands our understanding of mobile health consultations and provides important practical insights for facilitating deep physician–patient interactions and patient satisfaction online.

## Figures and Tables

**Table 1:** Related Works.

Information Form	Literature	Language Form	Key Variables	Key Findings
Information expression	This study	Language and paralanguage	Depth of interaction (content relevance was considered), emoticons, and inclusive language.	Findings: Emojis and inclusive language have a positive impact on patient satisfaction. The depth of doctor–patient interaction mediates this impact.
	2022; [32]	paralanguage	Pitch and Intensity.	Findings: The pitch of the physician’s voice has a positive effect on patient-perceived satisfaction, and the intensity has a negative effect on patient-perceived satisfaction, respectively, and the physician’s popularity moderates this effect. In addition, patient-perceived satisfaction affects subsequent economic returns for physicians.
	2020; [27]	paralanguage	Speech rate and average spectral centroid.	Findings: physician speech rate had a positive effect on patient satisfaction, and average spectral centroid had a negative effect on patient satisfaction. Physicians’ professional capital moderated this effect.
	2023; [7]	Language	Vocabulary richness, health-related terms, and emotional expressions.	Findings: Physician-level and individual-level language styles (vocabulary richness, health-related terminology, and emotional expressions) affect patient mood.
Information content	2019; [8]	Language and paralanguage	Depth of interaction (only the frequency of interaction was considered), response time, and service content.	Findings: Depth of interaction, physician service content, and response time significantly impacted patients’ decisions to continue consulting. Their impact on patient satisfaction varied over time.
	2020; [9]	Language	Information support, emotional support, physician responsiveness, and use of voice services or not.	Findings: The user’s perceived quality of service is influenced by the physician’s information support, emotional support, responsiveness, and use of voice service, in addition to the interaction effects between them.
	2015; [5]	paralanguage	Response speed, interaction frequency, and patient’s risk of disease.	Findings: Interaction frequency and response speed are important aspects related to patient satisfaction. In addition, patients’ risk of illness moderated their relationship.
	2022; [34]	Language and paralanguage	Frequency of interaction, message delivery method, and medical information.	Findings: Frequency of interaction, medical information, and message delivery method are three important aspects of online physician reviews bias. In addition, physician specialization varies to affect the effectiveness of voice messages.
	2020; [4]	Language	Patient’s activity; Physician’s informational support; Physician’s emotional support; Severity of patient’s disease.	Findings: Informational and emotional support significantly affected patient satisfaction, and the effect of emotional support was greater. The severity of the patient’s illness moderated the association between them.

**Table 2:** Definition of Interaction Depth.

Literature	Dimension	Research Field	Definition
2015; [41]	Frequency	Online learning	Frequency of participants interacting with the 3D virtual world.
2019; [8]	Frequency	Online healthcare	Number of interactions between physician and patient.
2020; [18]	Content	Online learning	Interactive content closely related to goals.

**Table 3:** Definition of Research Variables.

	Variables	Measurement and Description
Dependent variable	PatSatis	If the patient leaves a satisfactory evaluation, the code is 1 and the other is 0.
Independent and Mediating variables	Depth	Use content-relevant interactions to measure interaction depth.
	Inclusive	Inclusive language is measured by the number of “first-person plural” that a physician uses during counseling.
	Emojis	Frequency of emoji usage is measured by the number of emoticons used by the physician during counseling.
Control variables	RsT	The average response time of a physician is the average time difference between the physician’s response to the patient across all physician–patient interactions.
	QLen	The complexity of the patient’s question is measured using the total number of words asked by the patient during the counseling.
	D_Gender	The physician’s gender is a binary variable (1 for male physicians, 0 for female physicians).
	Price	Single counseling price.
	Total	Total number of patients received by physician online.
	GoodR	Physician’s online recommended value.
	D_Title	The physician’s professional title. Chief Physician, Associate Chief Physician, Attending Physician, and Physician are indicated as 4, 3, 2, and 1, respectively.
	HosLevel	The rank of the physician’s hospital (1 for tertiary hospitals, 0 otherwise).
	P_Active	The total number of examination pictures provided by the patient in the interaction.
	P_Civility	Total number of polite words used by patients.

**Table 4:** Descriptive Statistics.

Variable	N	Mean	Std	Min	Max
PatSatis	5064	0.807	0.390	0	1
Depth	5064	4.070	3.500	0	25.24
Inclusive	5064	0.110	0.460	0	7
Emojis	5064	0.520	1.350	0	19
HosLevel	5064	0.500	0.500	0	1
Ln(Total)	5064	8.460	1.330	4.530	11.52
GoodR	5064	5064	4.940	0.090	4.500
D_Title	5064	1.970	0.850	1	4
Ln(Price)	5064	1.540	1.210	0	6.080
Ln(QLen)	5064	4.630	0.930	0	7.670
D_gender	5064	0.480	0.500	0	1
RsT	5064	0.020	0.020	0	0.240
P_Active	5064	2.150	2.760	0	50
P_Civility	5064	1.690	1.730	0	24

**Table 5:** Regression Results.

	Model 1	Model 2	Model 3
HosLevel	−0.0074	−0.6410	−0.5216
	(1.9819)	(1.9420)	(1.9302)
Ln(Total)	0.1368	−0.1874	−0.1947
	(0.6521)	(0.6393)	(0.6353)
GoodR	9.1268	7.0232	6.3844
	(10.2176)	(10.0107)	(9.9496)
D_Title	−0.9977	−1.1112	−1.0318
	(0.8208)	(0.8041)	(0.7992)
Ln(Price)	0.5347 ***	0.4706 ***	0.4310 ***
	(0.0496)	(0.0488)	(0.0488)
D_Gender	−2.7371	−1.3965	−1.2793
	(2.0866)	(2.0463)	(2.0338)
RsT	15.2348 ***	16.0445 ***	16.3005 ***
	(2.5029)	(2.4526)	(2.4378)
Ln(QLen)	1.4865 ***	1.4261 ***	1.4165 ***
	(0.0500)	(0.0491)	(0.0489)
P_Active	0.1643 ***	0.1642 ***	0.1656 ***
	(0.0160)	(0.0157)	(0.0156)
P_Civility	0.0501 *	0.0299	−0.5216
	(0.0263)	(0.0258)	(1.9302)
Inclusive		1.3604 ***	1.3004 ***
		(0.0965)	(0.0962)
Emojis			0.3064 ***
			(0.0398)
FE Month	Yes	Yes	Yes
FE Physician	Yes	Yes	Yes
_cons	−48.1987	−34.9483	−31.8304
	(49.5114)	(48.5124)	(48.2166)
N	5064	5064	5064
R2	0.33	0.36	0.37

Note: Standard errors in parentheses. * *p* < 0.1, *** *p* < 0.01.

**Table 6:** Logistic Regression Results.

Variables	Model 1	Model 2
Depth		0.0575 ***
		(0.0145)
Inclusive	0.3198 **	0.2362 *
	(0.1273)	(0.1291)
Emojis	0.6059 ***	0.5845 ***
	(0.0824)	(0.0828)
HosLevel	−0.1785 *	−0.1516
	(0.0918)	(0.0922)
Ln(Total)	−0.1870 ***	−0.1787 ***
	(0.0331)	(0.0331)
GoodR	4.8030 ***	4.6613 ***
	(0.4063)	(0.4088)
D_Title	−0.0172	−0.0128
	(0.0501)	(0.0501)
Ln(Price)	−0.1584 ***	−0.1715 ***
	(0.0377)	(0.0379)
D_Gender	0.0361	0.0631
	(0.0810)	(0.0814)
RsT	8.4656 ***	6.9138 ***
	(2.4331)	(2.4577)
Ln(QLen)	0.0824 *	0.0021
	(0.0450)	(0.0495)
P_Active	0.0353 **	0.0288 *
	(0.0169)	(0.0171)
P_Civility	0.8248 ***	0.8446 ***
	(0.0444)	(0.0451)
_cons	−21.2444 ***	−22.0378 ***
	(2.0223)	(2.0074)
Sample Size	5064	5064
Pseudo R2	0.200	0.204

Note: Standard errors in parentheses. * *p* < 0.1, ** *p* < 0.05, *** *p* < 0.01.

**Table 7:** Hypothesis Testing Results.

Research Hypothesis	Result
H1: The effect of physicians’ use of inclusive language on patient satisfaction is positive.	Support
H2: The effect of physician use of emojis on patient satisfaction is positive.	Support
H3: The effect of depth of online interaction on patient satisfaction is positive.	Support
H4: The effect of physicians’ use of inclusive language on the depth of online interactions is positive.	Support
H5: The effect of physicians’ use of emojis on the depth of online interaction is positive.	Support

**Table 8:** Robustness Test Regression Results.

	First	Second	First	Second
	Inclusive	Depth	Emojis	Depth
	(1)	(2)	(3)	(4)
Inclusive_y	1.1623 ***			
	(0.0245)			
Inclusive		1.2651 ***		
		(0.1637)		
Emojis_y			1.1303 ***	
			(0.0216)	
Emojis				0.3797 ***
				(0.0642)
Contral Variables	Yes	Yes	Yes	Yes
FE Month	Yes	Yes	Yes	Yes
FE Physician	Yes	Yes	Yes	Yes
N	5064	5064	5064	5064
r2	0.4672	0.4047	0.6611	0.3894
Cragg-Donald Wald F statistic	2248		2750	

Note: Standard errors in parentheses. *** *p* < 0.01.

**Table 9:** Non-monotonic effects of emoji.

Variables	PatSatis
Emojis	0.7307 ***
	(0.0962)
Emojis^2^	−0.0379 ***
	(0.0098)
Inclusive	0.3207 **
	(0.1271)
Control Variables	Yes
Sample Size	5064
Pseudo R2	0.2012

Note: Standard errors are in parentheses. For demonstration purposes, we omit the display of control variables. ** *p* < 0.05, *** *p* < 0.01.

**Figure A1:**
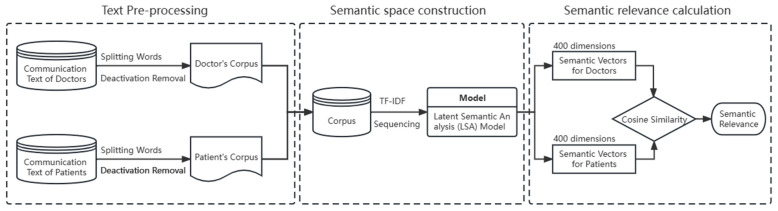
Semantic Relevance Model.

## Data Availability

Data sharing is not applicable to this article.

## Appendix A

We chose a technique called latent semantic analysis (LSA) as a tool to measure the content relevance of physician–patient interaction texts for several reasons: first, LSA allows the use of a vector form to represent the underlying semantics in documents of any length in a given semantic space. This facilitates the measurement of semantics in brief physician–patient interaction texts [67]. Second, LSA is widely used in academia, and the semantics mined through LSA are believed to have considerable similarity to the semantics understood by humans [68]. In particular, a recent study provides a preliminary validation of the feasibility of LSA applications in physician–patient communication scenarios [60].

We use Python to implement LSA and extract semantics from text, as shown in Figure A1. The key to correctly calculating the relevance of the content of two texts is how the researcher defines the semantic space. To avoid additional noise, this study chose the ac-quired dataset of physician–patient interactions rather than external texts to generate the semantic space. First, following the suggestion of Vrana et al., we classified all communication records of physicians and patients according to two subjects, physician and patient, and preprocessed the texts with word separation and deactivation removal as documents for LSA modeling [60]. Second, another key issue in LSA modeling is the selection of dimensions in the semantic space. Including too many dimensions may encompass too much noise, and conversely, including too few dimensions may lose some useful information. Since the semantic space of this study was constructed based on 14,986 physician–patient interaction passages and 35,433 unique terms referring to the practice of Gefen et al. [69], we chose to retain 400 dimensions after processing the bag-of-words representations of physician and patient texts into vectors for input to the SVD matrix decomposition by serialization and TF-IDF. Finally, as an output of the model, we used cosine similarity to calculate physicians’ and patients’ content relevance (SemR).

## Appendix B

To test the research hypothesis of this paper. We constructed three models based on the mediating model test approach.

The dependent variable of Equation (A1) was patient satisfaction, a dichotomous variable. This model was used to test the main effect of physician writing language style on patient satisfaction.

(A1) $${PatSatis}_{ijt}=\beta_{0}+\beta_{1}{Inclusive}_{ijt}+\beta_{2}{Emojis}_{ijt}+\delta {Control}_{ijt}+\varepsilon_{ijt},$$

Equation (A2) is an OLS regression model with physician and month-fixed effects, which tests the effect of a physician’s writing speech style on the depth of interaction. *i* in the model is the individual physician (range 1–337), *t* refers to the month (range 1–12), *j* is the current counseling, and $\varepsilon_{ijt}$ is the error term.

(A2) $${Depth}_{ijt}=\beta_{0}+\beta_{1}{Inclusive}_{ijt}+\beta_{2}{Emojis}_{ijt}+\beta_{3}{D\_Gender}_{ijt}+\delta {Control}_{ijt}+\tau_{1}{Physician}_{i}+\tau_{2}{Month}_{t}+\varepsilon_{ijt},$$

Finally, since the research model in this paper covers two different types of models, OLS regression and logit regression, their regression coefficients are not comparable, and the usual linear regression analysis would bring about the consequences of underestimation of mediating effects and skewed confidence intervals if used. Therefore, according to the research methodology [70,71], we constructed Equation (A3). Equation (A3) was used to test the mediating effect of interaction depth on physician writing language style and patient satisfaction.

(A3) $${PatSatis}_{ijt}=\beta_{0}+\beta_{1}{Depth}_{ijt}+\beta_{2}{Inclusive}_{ijt}+\beta_{3}{Emojis}_{ijt}+\delta {Control}_{ijt}+\varepsilon_{ijt},$$

## Appendix C

**Table A1:** Robustness Test OLS Regression Results.

	Model 1	Model 2	Model 3
Inclusive		1.357 ***	1.298 ***
		(0.100)	(0.099)
Emojis			0.319 ***
			(0.041)
Control Variables	Yes	Yes	Yes
FE Month	Yes	Yes	Yes
FE Physician	Yes	Yes	Yes
N	4557	4557	4557
R2	0.33	0.36	0.37

Note: Standard errors in parentheses. For demonstration purposes, we omit the display of control variables. *** *p* < 0.01.

**Table A2:** Robustness Test Logistic Regression Results.

Variables	Model 1	Model 2
Inclusive	0.3056 **	0.2254 *
	(0.1282)	(0.1302)
Emojis	0.6359 ***	0.6151 ***
	(0.0875)	(0.0800)
Depth		0.0516 ***
		(0.0153)
Control Variables	Yes	Yes
Sample Size	4557	4557
Pseudo R2	0.201	0.204

Note: Standard errors in parentheses. For demonstration purposes, we omit the display of control variables. * *p* < 0.1, ** *p* < 0.05, *** *p* < 0.01.

## Appendix D

**Table A3:** Correlation Analysis.

Variables	(1)	(2)	(3)	(4)	(5)	(6)	(7)	(8)	(9)	(10)	(11)	(12)	(13)
(1) Depth	1.000												
(2) Inclusive	0.240	1.000											
(3) Emojis	0.169	0.139	1.000										
(4) HosLevel	0.019	−0.000	−0.012	1.000									
(5) Ln(Total)	−0.020	−0.014	−0.012	−0.314	1.000								
(6) GoodR	0.101	0.068	0.070	−0.081	0.079	1.000							
(7) D_Title	−0.012	0.024	0.023	−0.198	0.286	−0.092	1.000						
(8) Ln(Price)	0.157	0.109	0.063	0.345	0.100	−0.025	0.109	1.000					
(9) Ln(QLen)	0.023	−0.052	−0.004	−0.133	0.103	0.057	−0.072	−0.101	1.000				
(10) D_gender	−0.073	−0.002	0.002	0.118	−0.066	0.067	−0.119	0.013	−0.037	1.000			
(11) RsT	0.425	0.127	0.079	0.090	0.035	0.029	0.061	0.208	−0.171	0.032	1.000		
(12) P_Active	0.221	0.064	0.036	0.096	−0.009	−0.007	0.025	0.231	−0.091	0.009	0.235	1.000	
(13) P_Civility	0.167	0.113	0.189	0.097	−0.019	0.041	0.024	0.177	−0.092	0.014	0.328	0.162	1.000
